# Effect of Low-Intensity Transcranial Focused Ultrasound Stimulation on Neuropathic Pain: Protocol for a Randomized, Single-Blind, Placebo-Controlled, 3-Arm, Parallel-Group Trial

**DOI:** 10.2196/95379

**Published:** 2026-08-14

**Authors:** Nobuhiko Mori, Yuhei Hoshikuma, Koichi Hosomi, Akihiro Yamamoto, Takeshi Shimizu, Hui Ming Khoo, Naoki Tani, Satoru Oshino, Haruhiko Kishima

**Affiliations:** 1 Department of Neurosurgery, Graduate School of Medicine, The University of Osaka, 2-2 Yamadaoka, Suita, Osaka, 565-0871, Japan, 81 6-6879-3652; 2 Hanwa Memorial Hospital, Osaka, Japan

**Keywords:** transcranial focused ultrasound stimulation, noninvasive brain stimulation, neuropathic pain, protocol, randomized controlled trial

## Abstract

**Background:**

Neuropathic pain (NP) remains difficult to manage because conventional pharmacological therapies often have limited efficacy and intolerable side effects. Noninvasive neuromodulation techniques have emerged as potential alternatives with fewer adverse effects. Meta-analyses of randomized controlled trials suggest that high-frequency repetitive transcranial magnetic stimulation over the primary motor cortex has analgesic effects; however, its efficacy is modest and restricted by the inability to stimulate deeper brain regions. Low-intensity transcranial focused ultrasound stimulation (TUS) is a novel approach that enables precise targeting of deep brain structures and modulation of neural activity. Although promising in preclinical and preliminary human studies, its long-term therapeutic efficacy for NP remains unknown.

**Objective:**

This trial aims to explore the therapeutic effects and safety of repeated TUS sessions in patients with NP.

**Methods:**

This randomized, participant-blinded, placebo-controlled, 3-arm, parallel-group clinical trial will enroll 39 participants with NP (pain duration ≥3 months; pain intensity ≥4 on the numerical rating scale). Participants will be randomly allocated to one of three groups: (1) TUS targeting the primary motor cortex, (2) TUS targeting the posterior superior insula, or (3) sham stimulation. Each participant will undergo weekly sessions for 8 weeks. The primary outcome is change in weekly average pain intensity scores recorded in a pain diary. Secondary outcomes include additional pain scales, quality of life measures, psychological assessments, quantitative sensory testing, motor cortical excitability, and adverse events.

**Results:**

The first participant was enrolled on April 30, 2025, and 20 participants had been enrolled as of February 28, 2026, with enrollment ongoing. The final analysis will be conducted after completion of follow-up and data verification.

**Conclusions:**

To our knowledge, this will be the first randomized controlled trial to examine the long-term therapeutic effects of repeated low-intensity TUS for NP. It will provide exploratory proof-of-concept data regarding its clinical efficacy and safety.

## Introduction

Neuropathic pain (NP) is defined by the International Association for the Study of Pain as “pain caused by a lesion or disease of the somatosensory nervous system” [1]. It often persists after the original injury has healed and is typically resistant to standard pharmacological treatments. Common forms include postherpetic neuralgia, painful diabetic neuropathy, phantom limb pain, spinal cord injury–related pain, and central poststroke pain. A nationwide Japanese epidemiological survey estimated the prevalence of NP at 3.2%, corresponding to approximately 3.5 million adults in Japan [2]. Despite available therapies, many patients report inadequate pain relief, and adverse effects frequently limit long-term adherence. First-line agents such as gabapentinoids, tricyclic antidepressants, and serotonin-norepinephrine reuptake inhibitors are recommended, but their effectiveness remains limited, with numbers needed to treat ranging from 4.6 to 8.9 [3]. Repetitive transcranial magnetic stimulation (rTMS), a noninvasive neuromodulation technique that targets the primary motor cortex (M1), has been investigated as an alternative. Systematic reviews and meta-analyses suggest that rTMS provides analgesic benefits [3-5], although the efficacy remains modest, with a number needed to treat of 4.2. In our previous clinical trials, rTMS showed limited benefit for pain overall, and subgroup analyses revealed particularly reduced effects for lower-limb pain, likely because rTMS cannot adequately stimulate deeper cortical regions such as the M1 foot area [6-8]. Because the M1 foot region and many other pain-related structures are located deep within the brain, further development of noninvasive therapeutic strategies capable of modulating these targets is needed.

In recent years, focused ultrasound has been increasingly developed and applied for a variety of purposes. This technology delivers ultrasound at frequencies above 20 kHz through the skull and can be directed to a localized brain region, allowing for intervention in deep brain structures. Magnetic resonance–guided focused ultrasound for thalamic thermal ablation is already used in clinical practice for the treatment of tremors, whereas ongoing studies are exploring its role in microbubble-mediated opening of the blood-brain barrier. More recently, low-intensity transcranial focused ultrasound stimulation (TUS), which operates at intensities below 100 W/cm^2^, has emerged as a noninvasive technique to modulate neural activity from outside the skull. Over the past two decades, animal studies have shown that TUS can alter motor-evoked potentials (MEPs) and regional cerebral blood flow [9]. Since its first application in humans in 2013, TUS has been reported to shorten reaction times in motor tasks, modulate somatosensory and visual-evoked potentials, and influence perception. The proposed mechanism involves ultrasonic acoustic pressure acting on ion channels through mechanoreceptors, thereby modulating neuronal activity [10].

TUS is a novel technique capable of exerting localized effects on deep brain structures without surgery. In contrast, rTMS can stimulate only superficial cortical regions when applied focally, and attempts to target deeper areas inevitably result in diffuse activation. To date, no rTMS device has been developed that allows for precise stimulation of deep brain structures. Potential therapeutic targets for NP include the M1 foot area, insula, cingulate gyrus, and thalamic nuclei. Although rTMS has been unable to adequately stimulate these regions, TUS may offer this capability. Thus far, only 2 studies of TUS in the context of pain have been conducted in healthy participants [11,12], and 1 single-arm study has been reported in patients with NP [13]. We previously performed a sham-controlled randomized crossover feasibility study of TUS in patients with NP (jRCTs052230116). However, to the best of our knowledge, no randomized controlled trial has yet evaluated the clinical efficacy of TUS for NP. For clinical translation, the so-called offline effects—sustained aftereffects that persist beyond the stimulation period—are considered essential. As with rTMS, repeated sessions, rather than a single session, are likely required to achieve meaningful therapeutic benefits.

Given the exploratory nature of this preliminary study, we selected 2 stimulation sites—M1 and the posterior superior insular cortex (PSI)—to assess their potential therapeutic effects. M1 was chosen based on extensive evidence from prior clinical studies using rTMS [3-5], whereas the PSI was selected because it is a major projection site of the spinothalamic tract and plays a key role in pain perception [14]. Furthermore, previous reports have shown that deep brain stimulation of the PSI can alleviate pain [15]. The aim of this study is to explore the efficacy and safety of weekly TUS administered over 8 weeks in patients with NP to provide proof-of-concept data for this therapeutic approach.

## Methods

### Trial Design

**Figure 1.**
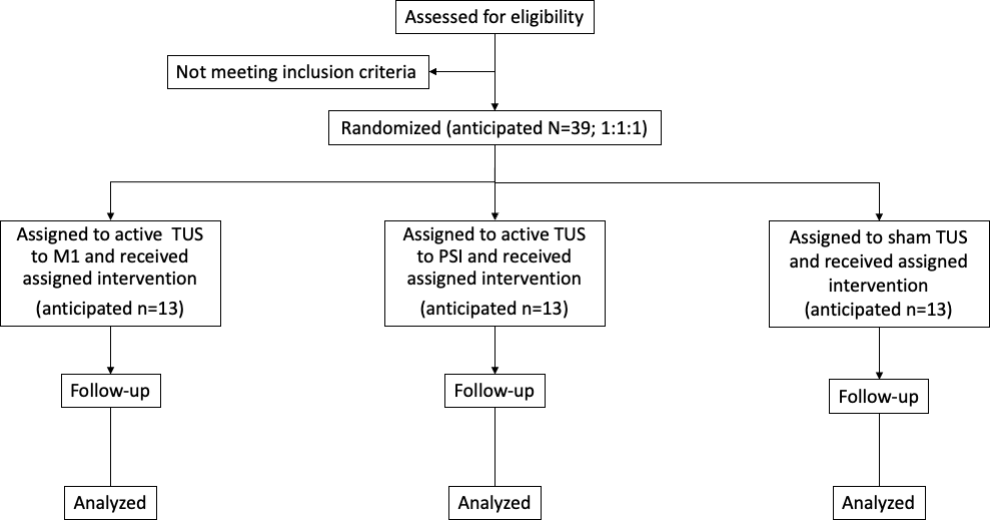
Flowchart of the study design. M1: primary motor cortex; PSI: posterior superior insular cortex; TUS: transcranial focused ultrasound stimulation.

This study is a randomized, single-blind (participant-blinded), placebo-controlled, parallel-group clinical trial conducted at the University of Osaka Hospital in Japan. Because pain is highly susceptible to placebo effects, a sham stimulation group was included as the control group to evaluate the superiority of active stimulation. A parallel-group design was selected instead of a crossover design owing to concerns about potential carryover effects and the risk of unblinding. To further explore potential differences in efficacy, 2 active stimulation sites were designated. The study flowchart is shown in Figure 1.

This study is an investigator-initiated clinical trial. Trial monitoring and auditing will be conducted by an independent academic research organization (Department of Medical Innovation, University of Osaka Hospital). Monitors will perform oversight in accordance with the monitoring plan to ensure data reliability and participant protection, verifying compliance with applicable laws, regulations, and the trial protocol, and will prepare monitoring reports. Data will be captured using an electronic data capture system (REDCap; Research Electronic Data Capture; Vanderbilt University), which minimizes input error, duplication, and missing data while ensuring security and transparency. This paper is based on protocol version 1.3 dated March 25, 2025.

### Participants

We will enroll patients aged 18 years or older with NP who meet the following inclusion criteria: (1) NP persisting for more than 3 months, (2) pain intensity score of 4 or more on the numerical rating scale (NRS) at screening, and (3) written informed consent to participate in this study. The definition of NP follows the International Association for the Study of Pain terminology [1], adopting a classification of probable or definite NP according to the NP grading system [16]. Exclusion criteria are as follows: (1) dementia with a Mini-Mental State Examination (MMSE) score of 23 or lower; (2) severe aphasia or cognitive dysfunction; (3) serious psychiatric disorder; (4) history of epileptic seizures; (5) use of an implantable stimulator, such as a cardiac pacemaker, except for implantable spinal cord stimulators; (6) metallic implants in the head, except for titanium products; (7) use of an implantable drug delivery system or implantable ventricular assist device; (8) pregnancy; (9) inability to complete the assessment questionnaires; (10) participation in any other clinical trial within 3 months prior to providing consent; and (11) any other condition deemed inappropriate for inclusion by the investigators. Participants will be recruited from outpatients of the Department of Neurosurgery at University of Osaka Hospital.

Participants will be withdrawn from the study if any of the following criteria are met after enrollment: (1) the investigator determines that continuing the study poses an unacceptable risk owing to the occurrence of an adverse event; (2) the participant withdraws consent or requests to discontinue participation; (3) it is discovered that the participant does not meet the eligibility criteria; (4) the participant is unable to complete the required assessments or visits as a result of personal circumstances, such as relocation; or (5) the investigator determines, for any other reason, that discontinuation of the intervention is appropriate.

### Randomization and Blinding

Participants will be randomly allocated in a 1:1:1 ratio to one of three groups: (1) active stimulation targeting M1, (2) active stimulation targeting PSI, or (3) sham stimulation. Because the primary outcomes rely on participant-reported subjective assessments, this study will adopt a single-blind design in which participants remain blinded to group allocation throughout the trial. Blinding of the investigators delivering the intervention is not feasible with the current device. As this is a preliminary study in which most outcomes are patient-reported, investigators are not blinded. To minimize expectation effects, all groups will undergo identical procedures, including treatment duration, interaction with study personnel, and auditory masking using white noise. The success of participant blinding will be evaluated at the end of the intervention period using a blinding assessment questionnaire.

Randomization will be conducted using a stratified permuted block method, with the underlying cause of NP (central or peripheral origin) as the stratification factor. The allocation table and procedure will be generated by an independent technician who is not involved in patient enrollment or outcome assessment. Assignment will be managed through the REDCap randomization module, which enhances secure handling of allocation information using password protection.

### Trial Schedule

**Figure 2.**
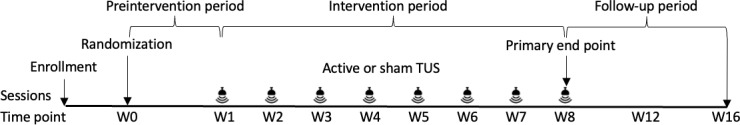
Trial schedule. TUS: transcranial focused ultrasound stimulation; W: week.

All patients who provide consent are assessed for eligibility by neurosurgeons specializing in pain management. Eligible participants will be randomly allocated to 1 of the 3 groups and undergo baseline evaluations, including demographic and clinical characteristics. The intervention is administered once a week for 8 weeks (Figure 2). This schedule was determined based on previous rTMS studies, which showed that intermittent stimulation—such as weekly sessions—provides significant pain relief for between 4 and 8 weeks or sessions, after which the effect tends to plateau [17-20]. Participants will be asked to record a pain diary from 1 week prior to the intervention until 1 week after completion at week 8. The NRS for pain and paresthesia (*shibire* in Japanese), along with the Short-Form McGill Pain Questionnaire version 2 (SF-MPQ-2) [21], will be assessed at baseline, immediately after each intervention, and at follow-up visits. Additional measures—including the Brief Pain Inventory (BPI) [22], EQ-5D-5L [23], Hospital Anxiety and Depression Scale (HADS) [24], Pain Catastrophizing Scale (PCS) [25,26], Pain Self-Efficacy Questionnaire (PSEQ) [27], MMSE, quantitative sensory testing (QST), motor cortical excitability, and the *shibire* questionnaire (validated by our group; A Yamamoto, unpublished data, October 2024) [28]—will be assessed at baseline and immediately after the final intervention at week 8. At the end of the study, Patient Global Impression of Change (PGIC) and blinding assessments will be collected. Adverse events will be monitored continuously from the start of the intervention until follow-up visits at weeks 12 and 16 or until study discontinuation. Potential adverse events associated with TUS will be explained to participants during the informed consent process. In addition, at each treatment session, participants will be asked whether they have experienced any new symptoms or deterioration. The detailed schedule is shown in Table 1.

**Table 1.** Schedule of enrollment, intervention, and assessment.

	Baseline assessment—week 0	Preintervention period	Intervention period								Follow-up period	
			Week 1	Week 2	Week 3	Week 4	Week 5	Week 6	Week 7	Week 8^a^	Week 12	Week 16
Eligibility screen	✓											
Demographic background	✓											
Intervention			✓	✓	✓	✓	✓	✓	✓	✓		
Pain diary^b^		✓	✓	✓	✓	✓	✓	✓	✓	✓		
Pain NRS^c^ and SF-MPQ-2^d^^,^^e^	✓		✓	✓	✓	✓	✓	✓	✓	✓	✓	✓
BPI^f^, EQ-5D-5L, HADS^g^, PCS^h^, and PSEQ^i^	✓									✓		
MMSE^j^	✓									✓		
Motor cortical excitability	✓									✓		
QST^k^^,^^l^	✓									✓		
*Shibire* questionnaire^m^	✓									✓		
*Shibire* NRS^e^^,^^m^	✓		✓	✓	✓	✓	✓	✓	✓	✓	✓	✓
PGIC^n^										✓		
Blinding										✓		
Adverse events			✓	✓	✓	✓	✓	✓	✓	✓	✓	✓
Malfunction of the device			✓	✓	✓	✓	✓	✓	✓	✓		

a If the intervention is discontinued, evaluations are performed at week 8.

b Pain diary is recorded from 1 week before the intervention until 1 week after its completion (week 8).

c NRS: numerical rating scale.

d SF-MPQ-2: Short-Form McGill Pain Questionnaire version 2.

e These measures are assessed immediately after each intervention.

f BPI: Brief Pain Inventory.

g HADS: Hospital Anxiety and Depression Scale.

h PCS: Pain Catastrophizing Scale.

i PSEQ: Pain Self-Efficacy Questionnaire.

j MMSE: Mini-Mental State Examination.

k QST: quantitative sensory testing.

l Assessed if the maximum pain site is located in the upper or lower extremities.

m Assessed if *shibire* is present. “*Shibire*” is a common Japanese term describing abnormal sensations, such as paresthesia, dysesthesia, or numbness, and may also refer to motor difficulty.

n PGIC: Patient Global Impression of Change.

### Sample Size Estimation

Given the exploratory nature of this study, the sample size was not intended to provide a definitive or fully powered estimate of treatment efficacy. As no previous clinical trials have examined the effects of repeated sessions of TUS in patients with chronic pain, the sample size calculation was based on a prior clinical trial of rTMS for NP [8]. In that study, the mean reduction in pain score immediately after the final intervention was 34.8 (SD 15.2) in the active stimulation group (n=15). Assuming that the sham group would experience half of this effect (mean reduction of 17.4) [3,8] and that both active TUS groups in our study would yield the same mean reduction (34.8), the SD was set at 15.2 for all groups. On the basis of these assumptions, a one-way ANOVA with an α of .05 and 80% power indicated that 37 participants would provide a reasonable basis for estimating potential between-group differences. Accounting for a 5% dropout rate, the final target sample size was set at 39 (13 per group). The anticipated dropout rate of 5% was based on a previous rTMS trial conducted by our group in patients with NP, in which the dropout rate was below 5% [8].

### Interventions

TUS will be administered using the NeuroFUS system (Sonic Concepts), which consists of a 4-element annular array transducer (CTX-500; Sonic Concepts) with a diameter of 60 mm and a fundamental frequency of 500 kHz. The transducer is powered by a programmable radiofrequency amplifier (TPO-203; Sonic Concepts), which controls the phasing of the 4 elements to adjust the sonication depth. A neuronavigation system (Brainsight; Rogue Research Inc) will be used to identify the stimulation site and precisely monitor and maintain the position and orientation of the transducer throughout all treatment sessions.

The stimulation target will be either M1, corresponding to the most painful region, or the PSI, both located contralateral to the pain. When the M1 hand area is selected, the motor hot spot identified through cortical excitability measurements using transcranial magnetic stimulation (TMS) will be used. The PSI will be localized on individual 3D magnetic resonance imaging (MRI) in the navigation system according to the previously reported “quadrant-within-a-quadrant” method [29]. For all other targets, stimulation sites will be determined on the individual MRI with reference to the Montreal Neurological Institute-Hospital (MNI) space standard atlas integrated into the navigation system [30]. The M1 foot target will be defined using the MNI coordinate (–7 or +7 mm; –27 mm and 59 mm) as the default location and subsequently adjusted based on each participant’s structural MRI. The central sulcus will be identified on axial images and traced medially, whereas the marginal ramus of the cingulate sulcus will be identified on the midsagittal image. The target will be placed in the anterior part of the paracentral lobule, immediately anterior to the central sulcus. If the default MNI coordinate falls within a sulcus, the target will be adjusted to the nearest cortex within the M1 foot area. Because the TUS device achieves optimal efficiency at a focal depth of 48.5 mm, an ultrasound gel pad of appropriate thickness (Echo Gel Pad; Yasojima Proceed Co., Ltd) will be placed between the transducer and the scalp to adjust the distance from the transducer surface to the stimulation target to within 43 to 54 mm. Stimulation parameters will be as follows: pulse duration of 10 ms, pulse repetition interval of 100 ms (10 Hz), pulse train duration of 10 seconds, pulse train repetition interval of 30 seconds, duty cycle of 10%, 20 trains, total stimulation duration of 580 seconds, and spatial peak pulse average intensity in free water of 30 W/cm^2^. The temporal stimulation pattern will be designed with reference to stimulation paradigms used in rTMS for refractory NP, consisting of intermittent 10-Hz pulse trains delivered over approximately 10 minutes [17,18,31]. Selected acoustic parameters, including stimulation frequency and intensity, will be informed in part by the human TUS protocol reported by Osada et al [32] using the same TUS device, although the temporal stimulation parameters will be modified for the present study. The stimulation intensity will be set within a range that has previously been applied without serious adverse events and remained within the safety limits recommended by the recent International Consortium for Transcranial Ultrasonic Stimulation Safety and Standards consensus statement [33]. The mechanical index, estimated using the TUS calculator [34] at a focus depth of 43 mm, was 1.32. On the basis of simulations reported by Osada et al [32], the maximum temperature increase will be estimated to be 0.5 °C in brain tissue and 0.8 °C in the skull. Extrapolating these values according to the effective sonication time used in the present study will yield conservative maximum estimates of 0.83 °C for brain tissue and 1.33 °C for the skull assuming no heat dissipation [32]. As the increase remained below 2 °C and the intervention time was less than 10 minutes, the thermal dose was estimated to be below 0.25 CEM43. The thermal index for the skull was calculated as 1.9. These values are within the safety thresholds recommended by the recent International Consortium for Transcranial Ultrasonic Stimulation Safety and Standards consensus on TUS safety [33].

Treatment will be administered with the participant seated comfortably in a reclining chair. Ultrasound gel will be applied to the scalp, gel pad, and transducer, with all visible air bubbles carefully removed. The transducer will then be affixed to the scalp via a gel pad to ensure complete contact. To maintain blinding, participants will not be shown the TUS device interface, and white noise will be delivered through earphones during stimulation to mask any device sounds. All interventions will be performed under the supervision of investigators who are also neurosurgeons specialized in neuromodulation. In the sham stimulation group, the transducer will be placed over M1, but no ultrasound will be emitted (0 W output), with the same device setup and procedures as in the active stimulation groups. Because TUS is not typically associated with distinct sensory perceptions during stimulation, participants are not expected to distinguish active from sham stimulation based on treatment sensation alone. A single sham condition will be used as a common control group for both active stimulation targets. Because participants will be assigned to only 1 treatment arm throughout the study and will not be exposed to multiple stimulation targets, differences in transducer location will not be expected to substantially affect participant blinding.

To minimize confounding effects, the initiation of new treatments for NP and alterations to existing regimens will, in principle, be prohibited from the time of enrollment until week 8. The use of rescue analgesics will be permitted during the study provided that it does not substantially deviate from the participant’s preintervention medication regimen. Participants will be instructed to maintain their existing analgesic treatment whenever possible throughout the study period. Exceptions may be permitted when required to ensure participant safety.

### Outcomes

#### Overview

The primary outcome is the change in weekly average pain intensity scores recorded in a pain diary using an 11-point NRS ranging from 0 (no pain) to 10 (worst imaginable pain). Participants will evaluate their average pain over the previous 24 hours daily at home. Weekly averages will be calculated, and changes from the baseline week to each postintervention week up to week 8 will be assessed. The primary end point is the change in weekly average pain scores from baseline to week 8. To assess clinically meaningful improvements, responder analyses will identify the proportion of participants achieving reductions of 2 points or more and 4 points or more [35] in weekly average pain scores at week 8 compared with baseline.

Secondary outcomes comprise patient-reported and physiological measures, enabling a multidimensional evaluation of pain, functional impact, psychological status, neurophysiological response, and patient perception over time. These include both longitudinal changes from baseline and postintervention assessments across the following domains.

#### Pain NRS

This will be assessed separately from the diary, with participants reporting their current pain on the same 11-point scale.

#### Short-Form McGill Pain Questionnaire Version 2

This questionnaire will evaluate the qualitative aspects of pain, with participants rating 22 items based on their current pain experience [21].

#### Brief Pain Inventory

This measure assesses pain presence, location, and intensity; analgesic use; and interference with daily life across 16 self-report items [22].

#### EQ-5D-5L

This instrument measures health-related quality of life across 5 domains [23].

#### Hospital Anxiety and Depression Scale

This scale screens for anxiety and depression using 14 items [24].

#### Pain Catastrophizing Scale

This scale assesses maladaptive cognitive responses to pain [25,26].

#### Pain Self-Efficacy Questionnaire

This questionnaire measures confidence in maintaining function despite pain [27].

#### Mini-Mental State Examination

This measure is administered by assessors to screen for cognitive impairment.

#### Quantitative Sensory Testing

This test is conducted using thermal and vibration stimulators (TSA-II and VSA-3000; Medoc) based on published methods [36]. Warm, cold, heat pain, cold pain, and vibration detection thresholds will be assessed on the volar forearm or calf of the painful side. Each session includes 2 practice and 4 formal measurements. These assessments will be performed only when the stimulation target is the upper or lower limb.

#### Motor Cortical Excitability

Evaluated using single- and paired-pulse TMS with a figure-8 coil of 70 mm, 2 Magstim 200^2^ stimulators, and a Magstim BiStim^2^ module. MEPs will be recorded from the first dorsal interosseous muscle on the painful side (Brainsight MEP module; Rogue Research Inc) elicited via contralateral M1 stimulation. Measures include resting motor threshold (RMT), short-interval intracortical inhibition, intracortical facilitation, and cortical silent period [37]. The motor hot spot is defined as the site eliciting the largest MEP and RMT as the lowest stimulation intensity evoking MEPs of 50 μV or more in at least 5 of 10 trials. Paired-pulse TMS will use a conditioning stimulus at 80% RMT and a test stimulus at 120% RMT, with interstimulus intervals of 2 ms (short-interval intracortical inhibition) and 15 ms (intracortical facilitation) plus control stimuli alone. Each stimulus will be delivered 10 times in a randomized order. The cortical silent period will be measured 10 times under 10% to 20% maximum voluntary contraction at 130% RMT, with an intertrial interval of at least 5 seconds.

#### Paresthesia (*Shibire*) NRS

Participants will rate intensity on a scale from 0 to 10. “*Shibire*” is a common Japanese term referring to abnormal sensations, such as paresthesia, dysesthesia, or numbness, and may also indicate motor difficulty, although it most often describes positive sensory symptoms such as paresthesia [28].

#### *Shibire* Questionnaire

This measure [28] evaluates the presence, quality, location, duration, intensity, and daily life impact of *shibire*.

#### Patient Global Impression of Change

This measure is administered at week 8, with participants rating overall perception of change on a 7-point Likert scale (“very much improved” to “very much worse”).

#### Adverse Events

These are defined as any unfavorable or unintended sign, symptom, or illness occurring in a participant regardless of causal relationship to the study. Worsening of preexisting conditions will also be considered adverse events.

#### Blinding Assessment

After the intervention, participants will be asked whether they believe they received real stimulation or sham stimulation or are unsure. Responses will be compared with actual treatment allocation using the Bang blinding index to assess the success of participant blinding [38].

### Statistical Analysis

Efficacy analyses will be performed on the full analysis set, defined as all randomized participants who received at least one stimulation session and had at least one postintervention outcome assessment (intention-to-treat analysis). A per-protocol set excluding participants with major protocol deviations will also be analyzed for sensitivity. Major protocol deviations will include violations of eligibility criteria identified after enrollment, withdrawal of consent, substantial nonadherence to the intervention protocol, and other deviations judged to have a significant impact on efficacy evaluation. The consistency of results between the full analysis set and the per-protocol set will be examined to evaluate the robustness of the primary findings. Any modifications or additions to the analyses after study initiation will follow an evaluation of their validity and potential impact, with corresponding amendments to the statistical analysis plan. Handling of outliers will be prespecified, and depending on the variable, either data transformation or statistical approaches robust to outliers will be applied.

Demographic characteristics and outcomes will be summarized descriptively, with longitudinal outcomes presented as line graphs over time. The primary analysis will use a linear mixed-effects model for repeated measures (MMRM) to evaluate changes in weekly average pain scores from the pain diary. The model will include change from baseline in weekly average pain scores as the response variable, with treatment group, time point, and their interaction as fixed effects and individual participants as a random effect. Baseline pain scores will be included as covariates. The primary treatment effects will be estimated using model-based contrasts comparing M1 stimulation with sham stimulation and PSI stimulation with sham stimulation at week 8. Multiplicity arising from these 2 primary comparisons will be controlled using the Dunnett adjustment. To identify time points showing significant changes from baseline, the Dunnett multiple comparison procedure will be used, with the baseline week serving as the control. The number and proportion of responders will be summarized by treatment group, and between-group comparisons will be performed using logistic regression models adjusted for baseline pain scores. Missing week 8 pain diary data will be imputed using the last observation carried forward method for responder analyses only. Secondary outcomes measured repeatedly, such as pain NRS and SF-MPQ-2, will also be analyzed using MMRM in the same way as the primary outcomes. Outcomes assessed only at baseline and week 8—including BPI, EQ-5D-5L, HADS, PCS, PSEQ, MMSE, QST, and MEP—will be evaluated using analysis of covariance, with change from baseline as the response variable, treatment group as a fixed effect, and baseline value as a covariate. Paresthesia-related outcomes will be analyzed using MMRM, analysis of covariance, or logistic regression depending on data type and timing. PGIC responses at week 8 will be summarized by treatment group and compared using the Fisher exact test. All analyses will be 2 sided, with *P* values below .05 considered statistically significant.

### Patient and Public Involvement

Patients and members of the public will not be involved in the design, recruitment, or conduct of this study.

### Ethical Considerations

This study will be conducted in accordance with the ethical principles of the Declaration of Helsinki and the Clinical Trials Act of Japan. The study protocol was reviewed and approved by the University of Osaka Clinical Research Review Board (approval number S24007). All protocol amendments will be reviewed by the committee. Participants will be provided with detailed information about the study by the investigators, and written informed consent will be obtained before enrollment. Participation is voluntary, and consent may be withdrawn at any time. To protect confidentiality, each participant who provides informed consent will be assigned an identification code, ensuring that individuals cannot be directly identified. The correspondence table linking codes to personal information will be securely stored to prevent any disclosure of personal data outside the trial.

The clinical study report will be made publicly available via the Japan Registry of Clinical Trials website following review by the Clinical Research Review Board and the institutional administrator. Findings will also be disseminated through scientific presentations and peer-reviewed publications, with strict adherence to confidentiality.

### Protocol Amendments

During peer review of this protocol manuscript, the Statistical Analysis section was revised to explicitly specify the prespecified primary treatment comparisons (M1 stimulation vs sham stimulation and PSI stimulation vs sham stimulation at week 8) and the planned multiplicity adjustment.

## Results

The trial was registered in the Japan Registry of Clinical Trials on December 27, 2024 (jRCTs052240227). Enrollment began on April 30, 2025. As of February 28, 2026, a total of 20 participants had been enrolled, and enrollment is ongoing. Data collection is expected to be completed by December 31, 2027. The final analysis will be conducted after completion of follow-up and data verification.

## Discussion

This study has several notable strengths. First, to our knowledge, it is the first randomized, participant-blinded, placebo-controlled clinical trial designed to exploratorily evaluate the sustained effects of TUS in patients with NP. While previous TUS studies in pain have been limited to healthy participants or single-arm exploratory designs, this trial systematically examines the clinical effects of repeated sessions over an 8-week period. Previous TUS studies investigating pain have largely been conducted in healthy participants. For instance, TUS of the posterior insula has been reported to alter pain perception, whereas stimulation of other insular regions modulated autonomic responses such as heart rate variability, suggesting functional specialization within the insular cortex [11]. On the basis of these findings, PSI was selected as one of the stimulation targets in the present trial. In addition, another study demonstrated that MRI-guided TUS targeting the anterior thalamus altered pain thresholds in healthy adults in a sham-controlled design, further supporting the ability of TUS to modulate central pain-processing networks [12]. A preliminary clinical study has also investigated the use of TUS in patients with NP. Shin et al [13] conducted a prospective single-arm exploratory trial using navigation-guided TUS targeting the anterior cingulate cortex in 11 patients with chronic NP. They reported significant reductions in pain intensity along with improvements in pain-related interference with daily life. However, because the study was conducted without a control group and involved a small sample size, the ability to draw definitive conclusions regarding clinical efficacy remains limited [13].

Second, TUS is a novel noninvasive neuromodulation technique that enables precise targeting of both superficial and deep brain structures using a navigation system. Systematic reviews and meta-analyses suggest that rTMS targeting M1 produces analgesic effects in NP [3-5]. However, conventional rTMS primarily stimulates superficial cortical regions and may have limited efficacy when targeting deeper brain areas such as the M1 foot region [6-8]. To overcome this limitation, deep TMS using H-coil systems has been developed, allowing for stimulation of deeper brain regions. Nevertheless, H-coil stimulation typically generates a broader and less focal electric field, resulting in stronger stimulation of superficial areas and reduced spatial specificity [39]. In contrast, TUS enables relatively focal modulation of both superficial and deep brain structures [9].

This study also has several limitations. First, it is designed as an exploratory trial; therefore, it may be underpowered to detect subtle between-group differences. Second, the single-blind design, in which only participants are blinded, may introduce a potential risk of performance bias because operators cannot be blinded owing to technical constraints of the device. However, the primary outcome of this study is based on patients’ self-assessment of pain intensity, and participant blinding is considered the most critical factor in minimizing bias. Therefore, the validity of blinding for the primary outcome is considered reasonably assured. Nevertheless, residual bias related to the lack of investigator blinding cannot be completely excluded, particularly for secondary outcomes requiring investigator involvement. In addition, as a single-center study, the generalizability of the findings may be limited.

In conclusion, this trial represents, to our knowledge, the first randomized controlled trial to investigate the sustained therapeutic effects of repeated TUS in patients with NP. The findings are expected to provide exploratory proof-of-concept evidence regarding the safety and clinical efficacy of this TUS intervention.

## Supplementary material

10.2196/95379 Checklist 1 SPIRIT (Standard Protocol Items: Recommendations for Interventional Trials) 2025 checklist.

## Abbreviations

BPI: Brief Pain Inventory
HADS: Hospital Anxiety and Depression Scale
M1: primary motor cortex
MEP: motor-evoked potential
MMRM: mixed-effects model for repeated measures
MMSE: Mini-Mental State Examination
MNI: Montreal Neurological Institute-Hospital
MRI: magnetic resonance imaging
NP: neuropathic pain
NRS: numerical rating scale
PCS: Pain Catastrophizing Scale
PGIC: Patient Global Impression of Change
PSEQ: Pain Self-Efficacy Questionnaire
PSI: posterior superior insular cortex
QST: quantitative sensory testing
REDCap: Research Electronic Data Capture
RMT: resting motor threshold
rTMS: repetitive transcranial magnetic stimulation
SF-MPQ-2: Short-Form McGill Pain Questionnaire version 2
TMS: transcranial magnetic stimulation
TUS: transcranial focused ultrasound stimulation

## References

[1] Loeser JD, Treede RD (2008). The Kyoto protocol of IASP Basic Pain Terminology. Pain.

[2] Inoue S, Taguchi T, Yamashita T, Nakamura M, Ushida T (2017). The prevalence and impact of chronic neuropathic pain on daily and social life: a nationwide study in a Japanese population. Eur J Pain.

[3] Soliman N, Moisset X, Ferraro MC (2025). Pharmacotherapy and non-invasive neuromodulation for neuropathic pain: a systematic review and meta-analysis. Lancet Neurol.

[4] Lefaucheur JP, Aleman A, Baeken C (2020). Evidence-based guidelines on the therapeutic use of repetitive transcranial magnetic stimulation (rTMS): an update (2014-2018). Clin Neurophysiol.

[5] O’Connell NE, Marston L, Spencer S, DeSouza LH, Wand BM (2018). Non-invasive brain stimulation techniques for chronic pain. Cochrane Database Syst Rev.

[6] Mori N, Hosomi K, Nishi A (2021). Exploratory study of optimal parameters of repetitive transcranial magnetic stimulation for neuropathic pain in the lower extremities. Pain Rep.

[7] Mori N, Hosomi K, Nishi A (2021). Difference in analgesic effects of repetitive transcranial magnetic stimulation according to the site of pain. Front Hum Neurosci.

[8] Hosomi K, Sugiyama K, Nakamura Y (2020). A randomized controlled trial of 5 daily sessions and continuous trial of 4 weekly sessions of repetitive transcranial magnetic stimulation for neuropathic pain. Pain.

[9] Sarica C, Nankoo JF, Fomenko A (2022). Human studies of transcranial ultrasound neuromodulation: a systematic review of effectiveness and safety. Brain Stimul.

[10] Darmani G, Bergmann TO, Butts Pauly K (2022). Non-invasive transcranial ultrasound stimulation for neuromodulation. Clin Neurophysiol.

[11] Legon W, Strohman A, In A, Payne B (2024). Noninvasive neuromodulation of subregions of the human insula differentially affect pain processing and heart-rate variability: a within-subjects pseudo-randomized trial. Pain.

[12] Badran BW, Caulfield KA, Stomberg-Firestein S (2020). Sonication of the anterior thalamus with MRI-guided transcranial focused ultrasound (tFUS) alters pain thresholds in healthy adults: a double-blind, sham-controlled study. Brain Stimul.

[13] Shin DH, Son S, Kim EY (2023). Low-energy transcranial navigation-guided focused ultrasound for neuropathic pain: an exploratory study. Brain Sci.

[14] Garcia-Larrea L, Mauguière F (2018). Pain syndromes and the parietal lobe. Handb Clin Neurol.

[15] Dongyang L, Cunha PH, Lapa JD (2026). Insula deep brain stimulation for neuropathic pain: a cross-over, randomized, sham-controlled trial. Neuromodulation.

[16] Finnerup NB, Haroutounian S, Kamerman P (2016). Neuropathic pain: an updated grading system for research and clinical practice. Pain.

[17] Mori N, Hosomi K, Nishi A (2024). Repetitive transcranial magnetic stimulation focusing on patients with neuropathic pain in the upper limb: a randomized sham-controlled parallel trial. Sci Rep.

[18] Attal N, Poindessous-Jazat F, De Chauvigny E (2021). Repetitive transcranial magnetic stimulation for neuropathic pain: a randomized multicentre sham-controlled trial. Brain.

[19] Quesada C, Pommier B, Fauchon C (2018). Robot-guided neuronavigated repetitive transcranial magnetic stimulation (rTMS) in central neuropathic pain. Arch Phys Med Rehabil.

[20] Kobayashi M, Fujimaki T, Mihara B, Ohira T (2015). Repetitive transcranial magnetic stimulation once a week induces sustainable long-term relief of central poststroke pain. Neuromodulation.

[21] Maruo T, Nakae A, Maeda L (2014). Validity, reliability, and assessment sensitivity of the Japanese version of the short-form McGill Pain Questionnaire 2 in Japanese patients with neuropathic and non-neuropathic pain. Pain Med.

[22] Uki J, Mendoza T, Cleeland CS, Nakamura Y, Takeda F (1998). A brief cancer pain assessment tool in Japanese: the utility of the Japanese Brief Pain Inventory--BPI-J. J Pain Symptom Manage.

[23] EuroQol Group (1990). EuroQol - a new facility for the measurement of health-related quality of life. Health Policy.

[24] Hatta H, Higashi A, Yashiro H (1998). A review of the reliability and validity of the Japanese version of the Hospital Anxiety and Depression Scale: results for women [Article in Japanese]. Jpn J Psychosom Med.

[25] Matsuoka H, Sakano Y (2007). Assessment of the cognitive aspects of pain: development of a Japanese version of the Pain Catastrophizing Scale and examination of its reliability and validity [Article in Japanese]. Jpn J Psychosom Med.

[26] Sullivan MJ, Bishop SR, Pivik J (1995). The Pain Catastrophizing Scale: development and validation. Psychol Assess.

[27] Adachi T, Nakae A, Maruo T (2014). Validation of the Japanese version of the Pain Self-Efficacy Questionnaire in Japanese patients with chronic pain. Pain Med.

[28] Yamamoto A, Hosomi K, Mori N (2025). An attempt to develop a questionnaire for "numbness" in the field of neurosurgery [Article in Japanese]. Jpn J Neurosurg.

[29] Ciampi de Andrade D, Galhardoni R, Pinto LF (2012). Into the island: a new technique of non-invasive cortical stimulation of the insula. Neurophysiol Clin.

[30] Mazziotta J, Toga A, Evans A (2001). A probabilistic atlas and reference system for the human brain: International Consortium for Brain Mapping (ICBM). Philos Trans R Soc Lond B Biol Sci.

[31] Bouhassira D, Jazat-Poindessous F, Farnes N (2024). Comparison of the analgesic effects of “superficial” and “deep” repetitive transcranial magnetic stimulation in patients with central neuropathic pain: a randomized sham-controlled multicenter international crossover study. Pain.

[32] Osada T, Nakajima K, Shirokoshi T (2024). Multiple insular-prefrontal pathways underlie perception to execution during response inhibition in humans. Nat Commun.

[33] Aubry JF, Attali D, Schafer ME (2025). ITRUSST consensus on biophysical safety for transcranial ultrasound stimulation. Brain Stimul.

[34] TUS calculator. Radboud University.

[35] Dworkin RH, Turk DC, Wyrwich KW (2008). Interpreting the clinical importance of treatment outcomes in chronic pain clinical trials: IMMPACT recommendations. J Pain.

[36] Rolke R, Magerl W, Campbell KA (2006). Quantitative sensory testing: a comprehensive protocol for clinical trials. Eur J Pain.

[37] Kobayashi M, Pascual-Leone A (2003). Transcranial magnetic stimulation in neurology. Lancet Neurol.

[38] Bang H, Ni L, Davis CE (2004). Assessment of blinding in clinical trials. Control Clin Trials.

[39] Roth Y, Amir A, Levkovitz Y, Zangen A (2007). Three-dimensional distribution of the electric field induced in the brain by transcranial magnetic stimulation using figure-8 and deep H-coils. J Clin Neurophysiol.

